# An introduction to family-centred services for children affected by HIV and AIDS

**DOI:** 10.1186/1758-2652-13-S2-S1

**Published:** 2010-06-23

**Authors:** Linda Richter

**Affiliations:** 1 Child, Youth, Family and Social Development, Human Sciences Research Council, South Africa

## Abstract

Family-centred services in the context of HIV/AIDS acknowledge a broad view of a "family system" and ideally include comprehensive treatment and care, community agencies and coordinated case management. The importance of family-centred care for children affected by HIV/AIDS has been recognized for some time. There is a clear confluence of changing social realities and the needs of children in families affected by HIV and AIDS, but a change of paradigm in rendering services to children through families, in both high-prevalence and concentrated epidemic settings, has been slow to emerge.

Despite a wide variety of model approaches, interventions, whether medical or psychosocial, still tend to target individuals rather than families. It has become clear that an individualistic approach to children affected by HIV and AIDS leads to confusion and misdirection of the global, national and local response. The almost exclusive focus on orphans, defined initially as a child who had lost one or both parents to AIDS, has occluded appreciation of the broader impact on children exposed to risk in other ways and the impact of the epidemic on families, communities and services for children. In addition, it led to narrowly focused, small-scale social welfare and case management approaches with little impact on government action, global and national policy, integration with health and education interventions, and increased funding.

National social protection programmes that strengthen families are now established in several countries hard hit by AIDS, and large-scale pilots are underway in others. These efforts are supported by international and national development agencies, increasingly by governments and, more recently, by UNAIDS and the global AIDS community.

There is no doubt that this is the beginning of a road and that there is still a long way to go, including basic research on families, family interventions, and effectiveness and costs of family-centred approaches. It is also clear that many of the institutions that are intended to serve families sometimes fail and frequently even combat non-traditional families.

## 

The idea that health and social services for children should be family centred is not new, but it has yet to take hold in the area of greatest need for millions of children worldwide - those affected by HIV and AIDS and related risk factors, whether these be poverty and migration or injecting drug use.

Family-centred services for children, rooted in the consumer-led movements of the 1960s, emerged towards the end of the twentieth century, initially in the fields of paediatric and geriatric care. For example, research on the adverse effects of separating young children from their caregivers led to policies that welcomed family members to be with their children during hospitalization and to participate in their children's care, especially if the clinical regime depended on continued active engagement of the family in the children's treatment and rehabilitation. As awareness of the embeddedness of the wellbeing of all individuals in social relationships and networks grew, family-centred services began to be accepted as a model for intervention [1].

Advocates of family-centred services for children point out that the family is the basic unit of care for children, with primary responsibility for the delivery of services to children and the greatest influence on a child's health and wellbeing prior to, during and subsequent to interventions by health and social welfare professionals. These convictions have driven fundamental changes in health legislation and practice in both the United States and elsewhere, according rights to families to be fully involved in the health and wellbeing of children [1,2].

The core concepts of family-centred care for children were first formally articulated in 1987 [3]. While more a philosophy than a set of prescribed practices, the most important concepts have been that:

1. Families are constant in the lives of children (and adults) while interventions through programmes and services are intermittent and generally short lived.

2. Families must be variously and inclusively defined.

3. Family-centred approaches are comprehensive and integrated.

4. Love and care within families, when recognized and reinforced, promote improved coping and wellness among children and adults.

Initial resistance by health professionals to the involvement of families in treatment were countered by evidence that revealed few, if any, ill effects of involving families, even in intensive care environments [4], as well as the many benefits of family participation. These include support for improved adherence, sensitive monitoring of changes in patient state, and extension of treatment and other services beyond the health facility [1,5].

Extensive experience of family-centred services has been gained, amongst others, in the care of children with chronic conditions [6], disabilities [7], child welfare [8], neonatology [9], and early interventions to promote the development of young children at risk [10].

## Family-centred services and children affected by HIV and AIDS

The importance of family-centred care for children affected by HIV/AIDS has long been recognized in the United States [1,11-15]. Twenty years ago, Carol Levine observed, "AIDS threatens the intimacy and acceptance that ideally undergird family relationships, while at the same time making them all the more powerful and necessary" [16]. Family-centred services in the context of HIV/AIDS acknowledge a broad view of a "family system" and ideally include comprehensive medical treatment, community agencies and coordinated case management [17].

Levine [16] speaks of family members as "individuals who by birth, adoption, marriage, or declared commitment share deep, personal connections and are mutually entitled to receive and obligated to provide support of various kinds to the extent possible, especially in times of need". The Task Force on AIDS and the Family concluded, "Families should be broadly defined to include, besides the traditional biological relationships, those committed relationships between individuals which fulfil the function of family" [18]. And, in 1994, the Global Programme on AIDS marked World AIDS Day under the banner, "AIDS and the Family".

The World AIDS Day Newsletter [19] pointed out that "any group of people linked by feelings of trust, mutual support and common destiny may be seen as a family. The concept need not be limited to ties of blood, marriage, sexual partnership or adoption. In this light, religious congregations, workers' associations, support groups of people with HIV/AIDS, gangs of street children, circles of drug injectors, collectives of sex workers ... may all be regarded as families".

Such definitions both respect traditional notions of family, as well as recognizing non-traditional forms of commitment arising from changes in reproductive biology, laws governing interpersonal obligations, acceptance of same-sex relationships, and deep association based on shared experience. In this sense, AIDS is a catalyst in expanding definitions of "family" to reflect the reality of contemporary life. More and more people live in non-traditional families, or "families of choice" [20], made up of some traditional family members, partners and friends [21].

There is a clear confluence of changing social realities and the needs of children in families affected by HIV and AIDS, but a change of paradigm in rendering services to children through families, in both high-prevalence and concentrated epidemic settings, has been slow to emerge. Rotheram *et al *[15] argue that the history of HIV, particularly in the United States, led to an individualistic focus that is proving hard to shift [22]. Despite a wide variety of model approaches, interventions, whether medical or psychosocial, tend to target individuals, not families [23-25].

Yet, when an individual is affected by HIV/AIDS, their family is inevitably affected [26,27]. Risk for infection is shared, as is apprehension about disclosure, stigmatization, ill-health and suffering, the costs and burdens of treatment, loss of income, and need for care and support. AIDS throws families into crisis, causing anxiety and stress wherever it occurs [28,29]. The full impact of HIV and AIDS, including its social and economic effects, is only appreciated when the family, and not only the individual, is the unit of analysis [30].

## Children affected by HIV and AIDS

Early into the new millennium, it became clear that an individualistic approach to children affected by HIV and AIDS was leading to confusion, and misdirecting, rather than amplifying, the global, national and local response [31]. There was an almost exclusive focus on orphans, defined initially as a child who had lost one or both parents to AIDS, to draw attention to the large number of children being made vulnerable by AIDS [32]. But this definition, with its focus on parental death, occluded appreciation of the broader impact on children exposed to risk in other ways and the impact of the epidemic on families, communities and services for children [33]. In addition, it led to narrowly focused, small-scale social welfare and case management approaches with little impact on government action, global and national policy, integration with health and education interventions, and increased funding.

It was under these conditions that the Joint Learning Initiative on Children and AIDS (JLICA) was launched in 2006. The JLICA was modelled on the Joint Learning Initiative on Human Resources for Health [34], as an independent, collaborative, cross-sectoral and multidisciplinary initiative with a finite goal [35]. The aim of the JLICA was to gather evidence, including about best practices, stimulate innovative thinking, and facilitate communication across disciplines and stakeholders in order to generate a set of high-level recommendations for the global community, governments, and international and local organizations. JLICA organized its work under four learning groups directed at topics suggested by the widely endorsed Framework for the Protection, Care and Support of Orphans and Vulnerable Children Living in a World with HIV and AIDS [36]: Strengthening Families; Community Action; Expanding Services and Protecting Human Rights; and Social and Economic Policies.

Spanning two years, the learning groups worked in a wide variety of ways, including by commissioning papers and through meetings, live and electronic debates, and a learning collaborative. The JLICA's final report was hailed as setting a new agenda for children [37], calling attention to the importance of families and family strengthening through family-centred services, economic assistance and social protection, and community support. Apart from reports generated by JLICA http://www.jlica.org, these arguments are set out in detail in Richter [38], Richter and Sherr [39], and Richter *et al *[40].

The death of a parent is an unspeakable loss for any child, an experience exacerbated by illness and suffering, potential loss of economic support, dislocation and separation from siblings. Adult deaths from AIDS continue to increase in the absence of antiretroviral treatment. But to focus only on orphans is to miss the bigger picture: 88% of so-called "orphaned" children have a surviving parent [38], and more than 90% of "orphans" live with close family [41,42]. Families were the first to respond to children affected by AIDS, both in the USA and in southern Africa [43,44], and have continued to be the vanguard of care and support for affected children.

Despite this, pitifully few resources and services are directed at bolstering and protecting this front line. Fewer than 15% of families caring for orphans and vulnerable children in 2007 were estimated to have received any assistance from external agencies [45]. It has taken equally long to recognize the role that communities play and the importance of strengthening these systems of care [46].

Surviving parents and families who take in children of relatives experience the stresses of increased dependency and, across the world, become poorer [47,48]. The death of working-age adults means the loss of jobs, livelihoods and skills, and additional care exacts heavy costs. The poorest families respond by cutting consumption: eating less and spending less on education and healthcare for other members of the family. All this critically affects the wellbeing of children [41,49].

The assumption that families are collapsing has led to a burgeoning of orphanages and other forms of institutional care drawing resources, even those intended to assist children affected by AIDS, away from families into expensive alternatives with known adverse effects on children's health and development [50]. While there is no question that families are under considerable strain, families are intimate social networks evolved for human care. As such, they continue to form, adapt and reconfigure, both throughout the family lifecycle and in response to external stressors [51,52]. Belsey [53] attests that it is the loss of family capital, in terms of resources, networks and reserves, that mediates the impact of HIV and AIDS on children and on the wider society. By his estimates, close to 60% of families in high-prevalence environments are directly affected by AIDS.

At its heart, AIDS can be thought of as a family disease. In high-prevalence environments, transmission occurs mainly in the family, between parents and children [54] and between partners and spouses [55]. Families are also on the front line of prevention [14], providing education and reinforcing risk reduction, especially among young people [56].

Levine [16] argues that the impact of AIDS on families, and the potential of families to be at the forefront of prevention, treatment and care, has not been fully appreciated, partly because people in high-risk groups, such as men who have sex with men, injecting drug users, sex workers, migrants and refugees, are inaccurately assumed to be isolated from family life. In concentrated epidemics, transmission from men who have sex with men (MSM), injecting drug users (IDUs) and sex workers spreads into families through concurrent heterosexual sex and sex with regular partners and spouses, and vertical transmission [57].

Among these extremely marginalized groups, families are also inevitably affected, whether in their roles as parents, spouses, partners, siblings, children or intimate others [58]. Despite the lack of attention to family factors in these populations, many MSM and IDUs are married [59], and most female sex workers have children and regular partners, in addition to clients. Families of these groups have been identified to be important for, among other things, prevention [60,61], disclosure [62,63], support [64], and treatment adherence [65].

## The way forward

The JLICA made strong recommendations regarding strengthening families through social protection and income transfers, on the one hand, and family strengthening through family-centred services on the other.

Social protection for families affected by HIV/AIDS is part of a groundswell of provision and demand for increased protection against destitution and improved social security, including for the poorest families in the poorest parts of the world [38,39,49,66]. National programmes are established in several countries hard hit by AIDS, including South Africa, Botswana, Mozambique, Namibia and Lesotho, and large-scale pilots are underway in, among others, Malawi, Zambia and Kenya. These efforts are supported by international and national development agencies, increasingly by governments [66] and, more recently, by UNAIDS and the global AIDS community [67].

The second prong of the response - family strengthening through family-centred services for children affected by HIV and AIDS - has yet to receive similar levels of endorsement and commitment. In response, the Coalition on Children Affected by AIDS (see http://www.ccaba.org), a network of child-focused foundations advised by researchers and advocates, started The Road to Vienna, an initiative to explore the nature of family-centred services, evidence for their feasibility and effectiveness, barriers to their expansion, and their relevance to especially marginalized populations. The initiative began with a meeting in Nairobi in late September 2009, piggy backed onto the first African Conference on "Promoting Family-Based Care for Children in Africa", organized by the African Network for the Prevention and Protection against Child Abuse and Neglect and its partners. Ten presentations were made on various aspects of familycentred services, including applications to prevention of mother to child transmission, antiretroviral (ARV) treatment for children, early child development services, and depression; five of these presentations appear as papers in this special issue (Bentancourt *et al*, Leeper *et al*, Bhana *et al*, Tomlinson, and Hosegood and Madhavan).

A second meeting was convened in Geneva in February 2010, in partnership with the International AIDS Society, to consider family-centred services for children and families of people in especially marginalized groups (MSM, IDUs, sex workers, and people currently or recently incarcerated). Seven presentations were made, together with a panel discussion, with strong participation from people representing affected groups. Three of these presentations appear as papers in this special issue (Beard *et al*, Solomon *et al*, and Sherr). What became clear from this meeting is the almost complete lack of research in this area, and a strong desire by people in marginalized groups to receive services to support their families and legal reform to help them to be good parents.

The rationale and available evidence for family-centred services for children affected by AIDS has not been brought together before. While there are very few clinical trials on family-centred services, DeGennaro and Weitz [68] make the point that individual components of family-centred services have been shown to be effective. These include home-based models of HIV voluntary counselling and testing [69], risk reduction following couple's counselling and testing [70], response to ARV treatment and adherence [71,72], prevention of mother to child transmission (PMTCT) [73], and child nutrition and education benefits of adult ARV programmes [74].

There are also clear costs for not adopting familycentred approaches to children affected by HIV and AIDS. These are especially evident in PMTCT programmes. For example, partner participation in programmes has been found to be associated with higher acceptance of post-test counselling, increased couple communication about HIV prevention, and increased use of ARVs [75]. Narrow pharmacological approaches are a lost opportunity for PMTCT to be the gateway to family-based prevention, care and treatment [73].

A piecemeal approach, tackling only one aspect of a complex multifaceted problem, also has the disadvantage that early successes may be reversed because later stage factors were not considered [76]. For example, eliminating HIV transmission to children is critical, but it does not eliminate risks to the mortality, morbidity and developmental progress of exposed but uninfected children [77,78].

## Conclusions

There are many different kinds of families, facing different kinds of challenges, and they will require different kinds of support. For example, Levine points out, "Because non-traditional families are more commonly socially and psychologically similar to the patient, having been deliberately formed around shared interests, they may be better equipped to respond to external pressures such as stigma, but not to the dependency and level of care occasioned by illness" [16]. But what seems unquestionable is that a family lens would significantly move forward our ability to understand contextual influences on HIV and AIDS prevention, treatment and care to ensure access by more people to services with better outcomes, and balance available resources across services, families and communities to achieve comprehensive and integrated care.

There is no doubt that this is the beginning of a road and that there is much to be done, including basic research on families, family interventions, and effectiveness and costs of family-centred approaches. It is also clear that many of the institutions that are intended to serve families (law, health care, social security and welfare, housing, work) sometimes fail and, importantly, frequently even combat non-traditional families. The latter may, at worst, be prosecuted for their lifestyle and lose custody of their children and, at least, be excluded from decisions about treatment, and be excluded from insurance benefits and/or home tenancy when a partner dies.

## Competing interests

The author declares that they have no competing interests.

## Authors' contributions

LMR conceived the review, conducted the search for materials and wrote the paper.

## Author information

LMR co-chaired Learning Group 1: Strengthening Families in the Joint Learning Initiative on Children and AIDS (JLICA) and is a member on the Committee of the Coalition on Children Affected by AIDS (CCABA). Executive Director, Child, Youth, Family and Social Development, Human Sciences Research Council, South Africa; Honorary Professor, Department of Psychology, University of KwaZulu-Natal, South Africa; Honorary Professor, Department of Paediatrics and Child Health, University of Witwatersrand, South Africa; Honorary Research Associate, University of Oxford, United Kingdom; Visiting Scholar, Harvard, University, United States.
